# The SNP rs460089 in the gene promoter of the drug transporter OCTN1 has prognostic value for treatment-free remission in chronic myeloid leukemia patients treated with imatinib

**DOI:** 10.1038/s41375-023-02109-2

**Published:** 2023-12-21

**Authors:** Katerina Machova Polakova, Ali Albeer, Vaclava Polivkova, Monika Krutska, Katerina Vlcanova, Nikola Curik, Alice Fabarius, Hana Klamova, Birgit Spiess, Cornelius F. Waller, Tim H. Brümmendorf, Jolanta Dengler, Volker Kunzmann, Andreas Burchert, Petra Belohlavkova, Satu Mustjoki, Edgar Faber, Jiri Mayer, Daniela Zackova, Panayiotis Panayiotidis, Johan Richter, Henrik Hjorth-Hansen, Magdalena Kamińska, Magdalena Płonka, Elżbieta Szczepanek, Monika Szarejko, Grażyna Bober, Iwona Hus, Olga Grzybowska-Izydorczyk, Ewa Wasilewska, Edyta Paczkowska, Joanna Niesiobędzka-Krężel, Krzysztof Giannopoulos, Francois X. Mahon, Tomasz Sacha, Susanne Saußele, Markus Pfirrmann

**Affiliations:** 1 Institute of Hematology and Blood Transfusion, Prague, Czech Republic; 2 Institut für Medizinische Informationsverarbeitung, Biometrie und Epidemiologie (IBE), Medizinische Fakultät, Ludwig-Maximilians-Universität, Munich, Germany; 3 Institute of Clinical and Experimental Hematology, 1st Medicine Faculty, Charles University, Prague, Czech Republic; 4 Department of Haematology and Oncology, University Hospital Mannheim, Heidelberg University, Mannheim, Germany; 5 UNIVERSITÄTSKLINIKUM FREIBURG Klinik für Innere Medizin I Schwerpunkt Hämatologie, Onkologie und Stammzelltransplantation, Freiburg, Germany; 6 Universitätsklinikum RWTH Aachen and Center for Integrated Oncology Aachen-Bonn-Cologne-Düsseldorf (CIOABCD), Aachen, Germany; 7 Onkologische Praxis Heilbronn, Heilbronn, Germany; 8 Universitätsklinikum Würzburg Medizinische Klinik und Poliklinik II, Würzburg, Germany; 9 University Hospital Marburg, Marburg, Germany; 10 4th Department of Internal Medicine - Hematology, University Hospital Hradec Kralove, Hradec Kralove, Czech Republic; 11 Translational Immunology Research Program and Department of Clinical Chemistry, University of Helsinki, Helsinki, Finland; 12 Hematology Research Unit Helsinki, University of Helsinki and Helsinki University Hospital Comprehensive Cancer Center, Helsinki, Finland; 13 Department of Hemato-oncology, Faculty Hospital and Faculty of Medicine and Dentistry, Palacký University, Olomouc, Olomouc, Czech Republic; 14 Internal Hematology and Oncology Clinic, Faculty Hospital Brno and Faculty of Medicine, Masaryk University, Brno, Czech Republic; 15 Hematology Clinic, National and kapodistrian University, Athens, Greece; 16 Dept. of Hematology, Oncology and Radiation Physics, Skåne University Hospital, Lund, Sweden; 17 Department of Hematology, St Olavs Hospital, Trondheim, Norway; 18 Department of Cancer Research and Molecular Medicine, Norwegian University of Science and Technology, Trondheim, Norway; 19 Department of Hematology, Jagiellonian University Hospital, Kraków, Poland; 20 Hematology and Transplantology Department, Medical University of Gdańsk, Gdańsk, Poland; 21 Hematology and Bone Marrow Transplantation Department, Medical Silesian University, Katowice, Poland; 22 Chair and Department of Hematooncology and Bone Marrow Transplantation Medical University of Lublin, Lublin, Poland; 23 Department of Hematology, Medical University of Lodz, Copernicus Memorial Hospital, Lodz, Poland; 24 Hematology Department, Medical University of Białystok, Białystok, Poland; 25 Department of General Pathology, Pomeranian Medical University, Szczecin, Poland; 26 Hematology, Oncology and Internal Medicine Department, Medical University of Warsaw, Warsaw, Poland; 27 Experimental Hematooncology Department, University of Lublin, Lublin, Poland; 28 Bergonie Institute Bordeaux, Inserm U1218 University of Bordeaux, Bordeaux, France

**Keywords:** Genetics research, Cancer genomics

## Abstract

Membrane transporters are important determinants of drug bioavailability. Their expression and activity affect the intracellular drug concentration in leukemic cells impacting response to therapy. Pharmacogenomics represents genetic markers that reflect allele arrangement of genes encoding drug transporters associated with treatment response. In previous work, we identified SNP rs460089 located in the promotor of *SLC22A4* gene encoding imatinib transporter OCTN1 as influential on response of patients with chronic myeloid leukemia treated with imatinib. Patients with rs460089-GC pharmacogenotype had significantly superior response to first-line imatinib treatment compared to patients with rs460089-GG. This study investigated whether pharmacogenotypes of rs460089 are associated with sustainability of treatment-free remission (TFR) in patients from the EUROpean Stop Kinase Inhibitor (EURO-SKI) trial. In the learning sample, 176 patients showed a significantly higher 6-month probability of molecular relapse free survival (MRFS) in patients with GC genotype (73%, 95% CI: 60–82%) compared to patients with GG (51%, 95% CI: 41–61%). Also over time, patients with GC genotype had significantly higher MRFS probabilities compared with patients with GG (HR: 0.474, 95% CI: 0.280-0.802, *p* = 0.0054). Both results were validated with data on 93 patients from the Polish STOP imatinib study. In multiple regression models, in addition to the investigated genotype, duration of TKI therapy (EURO-SKI trial) and duration of deep molecular response (Polish study) were identified as independent prognostic factors. The SNP rs460089 was found as an independent predictor of TFR.

## Introduction

A genetic background that affects drug transportation through the plasma membrane of the cells can influence drug availability and treatment response. The activity and number of transporters are important parameters for absorption and drug distribution through the cells of gastrointestinal tract and for targeting cancer cells. Solute carrier (SLC) proteins facilitate drug entry into the cells, whereas ATP binding cassette (ABC) transporters facilitate drug excretion. Several studies investigated transporters in relation to the therapy response of patients with chronic myeloid leukemia (CML). Imatinib, the first tyrosine kinase inhibitor approved for therapy of CML more than 20 years ago, is a known substrate of the transporters ABCB1 (MDR1, P-glycoprotein) [1, 2] and ABCG2 (BCRP) [3]. The most studied SLC transporter in relation to CML outcome on imatinib therapy was OCT1 (SLC22A1). It was shown that the major molecular response (MMR, i.e. ≤0.1% *BCR::ABL1*^IS^; IS – International Scale) was frequently observed during imatinib treatment in patients with increased OCT1 activity [4, 5]. In contrast, a minor contribution of OCT1 to the imatinib transport was found by Hu et al. [6]. A significantly higher affinity of imatinib was found to SLC transporters OATP1A2, OATP1B3 and OCTN2 compared to OCT1, OCT2, OCT3, OAT1, OAT2, OAT3 and OCTN1 [6]. Monitoring of the gene expression of imatinib transporters OCT1 or ABCB1 was not found applicable as markers of resistance to imatinib because their expressions significantly vary among the cell types that are present in samples of bone marrow or peripheral blood of CML patients [7, 8].

Several studies have shown an association of the response to imatinib, and single nucleotide polymorphisms (SNPs) found in coding regions of the selected SLC and ABC genes; M420del and rs683369 in OCT1 [9, 10], rs2032582, rs1128503 and rs60023214 in ABCB1 [11–13].

In our previous work, the large next generation sequencing screening of proximal promotor regions of 19 genes encoding SLC (*n* = 15) and ABC (*n* = 4) transporters with annotated function of drug transporters revealed that SNP rs460089 (located in the promotor region of SLC22A4) is significantly associated with response to imatinib first line therapy in CML patients in the European population [14]. Patients with rs460089-GC genotype (further in the text referred as GC) had significantly higher probability of sustained MMR achievement as compared with patients with rs460089-GG genotype (further in the text referred as GG). The clinical relevance of rs460089-CC genotype (further in the text referred as CC) could not be reliably evaluated due to the low frequency of this genotype in the population (around 10%). The SNP rs460089 is in the promotor region of the gene encoding OCTN1 (SLC22A4) transporter. The rs460089 is annotated as the regulatory locus within the conserved region for binding of transcription factors. Moreover, the rs460089 is in highly significant linkage disequilibrium with 7 other regulatory SNPs located in introns of *SLC22A4* (*n* = 3) and *SLC22A5* (OCTN2) (*n* = 3), as well as in one locus located downstream of the *SLC22A4*. The rs460089 alleles perfectly predict the alleles of the 7 regulatory loci [14]. Interestingly, the SNP rs460089 is in the linkage disequilibrium of the SNPs found in *SLC22A4* and *SLC22A5* genes as potential predictors of time to progression of gastrointestinal stromal tumors in patients with unresectable/metastatic form that are treated with imatinib [15].

This study investigated differences in the outcome after TKI cessation according to the genotypes of the SNP rs460089 in patients from the EUROpean Stop Kinase Inhibitor (EURO-SKI) clinical trial with first-line imatinib treatment [16]. We hypothesized that the GC genotype ensures lethal intracellular concentration of imatinib and more effectively eliminates CML cells compared to GG genotype, which impacts the probability of maintaining MMR after treatment discontinuation.

## Material and methods

### Samples

DNA samples were available from 301 EURO-SKI patients from participating centers from Germany, Czech Republic, Sweden, Finland, Norway and Greece. All these patients were treated with imatinib first-line before inclusion into the EURO-SKI study. The inclusion criteria included major type of *BCR::ABL1* transcript, TKI therapy for at least 3 years and at least 1 year of duration of deep molecular response (DMR) at least MR^4^ as defined by Cross et al. [17]. Detailed inclusion criteria are described in Saussele et al. [16]. This group of patients served as a learning sample for prognostic factor evaluation. All patients gave their informed consent for this work that required DNA isolated from peripheral blood during the study.

A validation sample was based on 103 patients from Polish academic centers treated with imatinib in the first line and entering the TFR phase after a minimum duration of 2 years DMR. The patients were routinely followed according to the European LeukemiaNet (ELN) recommendations. The criteria for the attempt of imatinib discontinuation were aligned with those described above for the learning sample. Patients discontinued imatinib therapy after signing the informed consent and local ethics committee approval. Patients were registered in the “Polish Stop Imatinib” web-based database on Hematoonkologia.pl, which was coordinated by the Polish Adult Leukemia Group (PALG). The RQ-PCR method standardized according to the ELN recommendations was used for molecular monitoring of treatment-free remission (TFR), i.e., MMR maintenance in ELN-certified laboratories [17]. *BCR::ABL1* transcript level monitoring on the IS was performed at imatinib discontinuation, in four-week intervals in the first six months after that, in six-week intervals in the next six months, and continuously in 3-month intervals subsequently. In the case of MMR loss, imatinib was reintroduced within four weeks from blood collection for the RQ-PCR test, which was then repeatedly performed in four-week intervals until the re-achievement of MMR, and in three-month intervals thereafter.

### Genotyping analysis

Sanger Sequencing of the SNP rs460089 described previously was performed in the learning cohort [14]. Moreover, the TaqMan SNP genotyping assay (ThermoFisher Scientific; Waltham, MA, USA) on real-time PCR platform (StepOnePlus, ThermoFisher Scientific) for analysis of rs460089 was tested and validated as a quick and cheap screening approach. The custom TaqMan SNP genotyping assay designed for the allele discrimination of the rs460089 failed. Due to the high GC content in the sequence surrounding the SNP, the allelic discrimination was poor. Therefore, the linkage disequilibrium (LD) analysis was performed using the LDlink web-based applications of the National Cancer Institute to search for the SNPs with significantly high LD [18]. Three SNPs in high LD with rs460089 were identified (Table S1). According to the in-silico analysis of parameters of the sequence surrounding a particular SNP for allelic discrimination analysis, the SNP rs156322 was selected. According to LDlink tool, alleles of the rs156322 [C/T] perfectly predict alleles of the rs460089 [C/G] in the European population. Thus, rs156322-T allele corresponds to rs460089-G allele and rs156322-C corresponds to rs460089-C allele.

The 10 µl PCR reaction for the allele discrimination analysis included: 5 µl of 2x TaqMan genotyping master mix, 0.5 µl of 20x Drug metabolism genotyping assay mix (Thermofisher Scientific) and 1.5 µl of DNA (10–15 ng/ µl). PCR conditions were applied according to the recommendations of the manufacturer as follows: 95 °C for 10 min following 50 cycles of 95 °C for 15 s and 60 °C for 90 s. Three Ph positive cell lines observed from DSMZ collection were used as positive controls. Each cell line carried a different genotype; MEG01 = CC, CML-T1 = GC and KCL-22 = GG. The evaluation of detected alleles was performed using the software provided by the manufacturer of the StepOne Plus instrument (Thermofisher Scientific). Sequencing analysis (more detailed in Supplementary methods) was performed for validation of the TaqMan SNP genotyping assay as previously [14].

### Statistical analysis

The additive model was applied for analysis of the SNP rs460089 genotypes “GG”, “Gc”, and “cc” (where “c” represents the minor allele) as three different numbers. This implies that the contribution of genotype “Gc” to the phenotype is different from “GG” and “cc”. From a biological perspective, this coding is reasonable, as the phenotypes of living organisms are not strictly “black and white”. Moreover, since we were analysing the regulatory SNP in the promoter region, all genotypes should be analysed separately due to possible transvection, an epigenetic mechanism involving the interaction between an allele on one chromosome and the corresponding allele on the homologous chromosome. For the proportions of molecular-relapse-free survival (MRFS) at 6 months (binary endpoint), the corresponding 95% confidence interval (CI) was estimated according to Wilson [19]. For reason of comparability between univariate and multiple models, the odds ratio (OR) was always calculated using logistic regression. Probabilities of MRFS over time and by 18 months were calculated with the Kaplan-Meier method. For comparisons of MRFS probabilities of genotype groups over time (time-to-event endpoint), the hazard ratios and p values were taken from the Cox model. However, since the hazards changed due to much fewer events after 6 months, the influence of the genotype groups on MRFS probabilities was also considered with the Cox cure model [20, 21]. Apart from the genotype of the SNP rs460089, candidate variables for prognostic influence on MRFS probabilities in all multiple models were sex, age at TKI discontinuation, TKI treatment duration before discontinuation of any therapy, duration of DMR before TKI discontinuation, and the time between start of TKI and the first observation of DMR. The EURO-SKI patients finally eligible for analyses formed the learning sample. Polish patients, not part of the EURO-SKI trial comprised a validation sample. The significance level of the two-sided *p* values was 0.05. All calculations were done with SAS 9.4.

## Results

### Validation of the TaqMan genotyping analysis

DNA samples of 301 EURO-SKI patients were sequenced for the SNP rs460089. The same samples were also genotyped using the TaqMan genotyping assay. Data observed from genotyping analysis were consensual with sequencing data in 297/301 EURO-SKI patients. In 4/301 patients the GG homozygote in rs460089 was identified by the TaqMan genotyping assay using the marker rs156322, but direct sequencing of the rs460089 identified GC genotype. The alleles of these 4 patients were identified as TT homozygotes in the rs156322, but one T allele segregated with C allele not with G allele of the rs460089. Two patients were Asian, one patient was from Greece and one from Germany, but regarding ancestors both of unknown origin. Using the LDlink it was found that the correlation of the alleles of the SNPs is lower in Asian population with R^2^ 0.969 compared to European population with R^2^ = 0.981.

The validated TaqMan genotyping assay was then applied to 103 Polish patients all of the European origin.

### Frequency of pharmacogenotypes in the EURO-SKI and the Polish sample

Frequency of pharmacogenotypes in 301 EURO-SKI patients was 48% for GG (*n* = 144), 42% for GC (*n* = 125) and 11% for CC (*n* = 32). In the Polish sample (*n* = 103), the GG genotype was present in 46 patients (45%), GC in 44 (43%), and CC in 13 (13%) patients. The allele frequency 68% for G and 32% for C of altogether 404 patients from both samples corresponded exactly with the allele frequency of European population for the rs460089 [14].

### Pharmacogenotypes were significantly associated with the probabilities of molecular relapse-free-survival after TKI stop

In addition to the inclusion criteria defined for the prognostic analysis in the EURO-SKI trial, all patients with interferon-alpha pre-treatment were excluded [16]. The observed effect of interferon-alpha on the response of CML patients in EURO-SKI could mask the influence of the studied SNP on MRFS. Of 301 EURO-SKI patients, firstly, 183 patients fulfilled the inclusion criteria for our analysis. Data on sex, duration of TKI treatment and of DMR, age at time of TKI discontinuation as well as molecular status at 6 months after discontinuation were available for 178/183 patients [22]. After an update in 2020, two more patients with interferon-alpha pre-treatment and one patient with unknown transcript were excluded. Finally, molecular status at 6 months was available for 176/180 patients. These 176 patients comprised the learning sample.

The GG genotype was identified in 96 patients (55%), GC in 62 (35%), and CC in 18 (10%) of 176 analysed patients (Table 1). Median age was 60 years, 49% of the patients were female.

**Table 1:** Patient Characteristics.

	Learning sample (*n* = 176)	Validation sample (*n* = 93)
SNP rs460089 genotype, number (percentage)		
GC	62 (35)	39 (42)
GG	96 (55)	41 (44)
CC	18 (10)	13 (14)
Gender, females, number (percentage)	86 (49)	52 (56)
Median age at TKI stop, years (range)	60 (23–84)	61 (23–83)
Median duration of TKI therapy before stop, years (range)	6.8 (3.1–13.5)	9.7 (3.1–14.4)
Median duration of DMR before TKI stop, years (range)	4.1 (1.1–12.8)	6.8 (2.0–11.5)
Median duration of TKI therapy before DMR, years (range)	1.7 (0.3–9.5)	1.7 (0.1–10.8)
Transcript type, number (percentage)*		
e13a2	39 (26)	24 (26)
e14a2	94 (62)	51 (55)
e13a2 + e14a2	18 (12)	18 (19)

*For 25 patients, the information “major breakpoint” but no transcript type was available.

*TKI* tyrosine kinase inhibitor, *DMR* deep molecular response.

Out of 176 patients, 105 (60%, 95% CI: 52–67%) maintained MMR 6 months after TKI stop. Most beneficial for MMR maintenance was genotype GC (73%, 95% confidence interval (CI): 60–82%), followed by CC (61%, 95% CI: 39–80%) and GG (51%, 95% CI: 41–61%). Overall, the SNP rs460089 was associated with MMR maintenance after TKI stop (*p* = 0.0286) with a significantly higher odds ratio (OR) for maintenance for GC genotype vs. GG (OR = 2.539, 95% CI: 1.278–5.045, *p* = 0.0078) but not for CC vs. GG (OR = 1.507, 95% CI: 0.539–4.216, *p* = 0.4343). Using CC as reference group, the OR for MMR maintenance was not significantly different with GC genotype (OR = 1.684, 95% CI: 0.539–4.216, *p* = 0.3527).

**Fig. 1:**
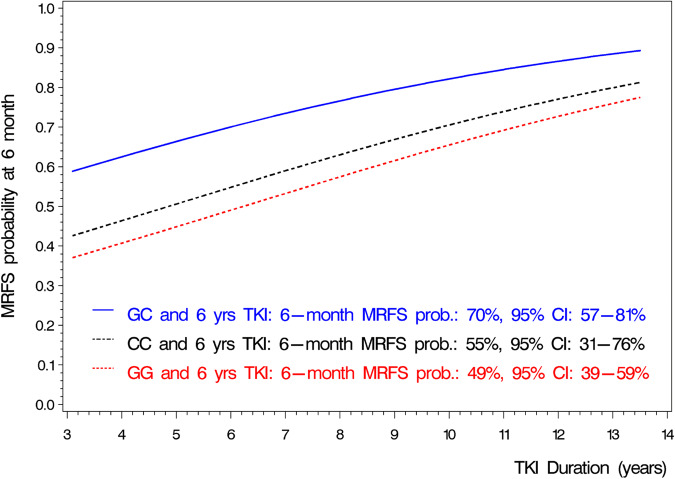
Probabilities for 6-month MMR maintenance for the combination of genotype group and duration of TKI treatment. In combination with the intercept –1.0564 (95% CI: −2.0198; −0.0930, *p* = 0.0316), the linear predictor (LP) is given by LP = −1.0564 + 0.8880 [if genotype = GC] + 0.2320 [if genotype = CC] + 0.1697 * [TKI duration in years (rounded to one decimal) before treatment stop]. The probability in Fig. 1 is then calculated by 1 / [1+exp(-LP)]. For the regression coefficients 0.8880, 0.2320, and 0.1697, the 95% CI were (0.1899;1.5860, *p* = 0.0127), (−0.8239; 1.2880, *p* = 0.6667), and (0.0337; 0.3056, *p* = 0.0144), respectively. MMR major molecular response, TKI Tyrosine kinase inhibitor, CI confidence interval, YRS years, Prob. probability.

Of the 176 patients, 150 (85%) had solely first-line imatinib before treatment stop. Twenty-six patients had second-line dasatinib (*n* = 14) or nilotinib (*n* = 12) before TKI discontinuation. Treatment change was only due to intolerance of imatinib. Significant associations between the number of TKI treatments and either genotype or maintenance of MMR were not observed. Only duration of TKI treatment was significant (OR = 1.185, 95% CI: 1.034–1.357, *p* = 0.0144) when added to genotypes in multiple logistic regression analysis. Longer duration of TKI treatment markedly increased the probability to maintain MMR 6 months after TKI stop while the overall association of the SNP rs460089 with MMR maintenance remained significant (*p* = 0.0442). The OR of GC vs. GG was slightly modified to 2.430 (95% CI: 1.209–4.884, *p* = 0.0127). Probabilities for 6-month MMR maintenance for the combination between genotype group and duration of TKI treatment are given in Fig. 1. In the EURO-SKI trial, patients had a significantly higher chance to maintain MMR at 6 months if TKI treatment duration had been more than 5.8 years. With 6 years of treatment duration, the probabilities to be at least in MMR 6 months after TKI discontinuation were 70% (95% CI: 57–81%) for the GC genotype, 55% (95% CI: 31–76%) for the CC, and 49% (95% CI: 39–59%) for the GG genotype (Fig. 1).

**Fig. 2:**
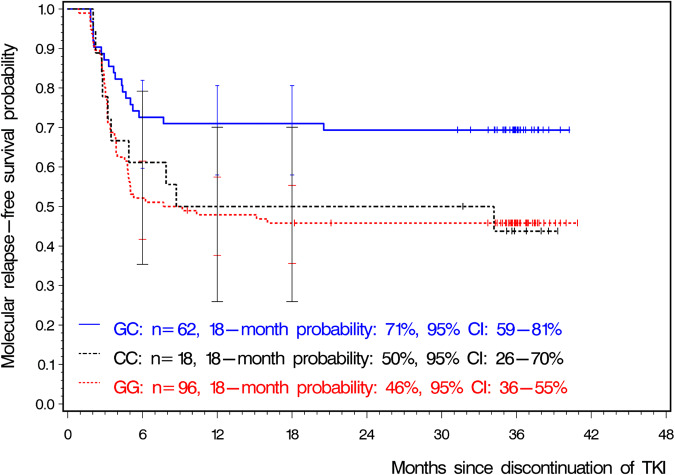
Probabilities of molecular relapse-free survival in 176 patients of the learning sample for each genotype of SNP rs460089. Bars at 6, 12, and 18 months indicate the upper and lower 95% CIs. CI confidence interval, TKI Tyrosine kinase inhibitor.

Median observation time was 36 months. Minimum observation time for patients without molecular relapse was 10 months. Ten patients experienced loss of MMR after 6 months. MRFS probability by 18 months was 55% (95% CI: 47–62%). SNP rs460089 was significantly associated with MRFS probabilities after TKI stop (*p* = 0.0147, Fig. 2). Patients with GC genotype had significantly higher MRFS probabilities compared with patients with GG (HR: 0.474, 95% CI: 0.280–0.802, *p* = 0.0054) but not with CC genotype (HR: 0.484, 95% CI: 0.225–1.042, *p* = 0.0635). In relation to genotype GG, GC was overall also significantly more favorable when the Cox cure model was applied. The logistic part of the model led to an OR of 0.373 (95% CI: 0.190-0.731, *p* = 0.0041) expressing a lower proportion of MMR losses. In contrast, the HR was not significantly lower (HR: 0.922, 95% CI: 0.542–1.567, *p* = 0.7632). The survival part of the Cox cure model addresses the distribution of the event times of those who experienced an event. The non-significant result means, if loss of MMR was observed, event times were not significantly earlier or later for patients with GC compared with GG. This was in line with the pattern of the Kaplan-Meier curves in Fig. 2. Using a multiple Cox cure model adjusting for other variables significant either in the logistic part (duration of TKI treatment) or the survival part (age at TKI discontinuation), the picture did not change: Compared with GG, the OR of GC for MMR loss was 0.373 (95% CI: 0.185–0.752, *p* = 0.0058) and the HR presenting the distribution of the times of MMR loss was 0.911 (95% CI: 0.536–1.559, *p* = 0.7307).

In 25 patients, a major breakpoint (p210) was confirmed but the transcript type remained unknown (Table 1). For this reason, transcript type was not initially considered in multiple models. Most beneficial with 65% MMR maintenance at 6 months was transcript type e14a2 (95% CI: 55–74%), followed by the appearance of both types, 61%, (95% CI: 39–80%) and e13a2, 44%, (95% CI: 29–59%). Overall, the association of transcript type with MMR maintenance at 6 months after TKI stop was not significant (*p* = 0.0788). Neither was transcript type significant when included in multiple logistic models. Considering loss of MMR as a time-to-event variable, patients with transcript type e14a2 had significantly higher MRFS probabilities compared with patients with e13a2 (HR: 0.479, 95% CI: 0.287–0.799, *p* = 0.0048) but not with both transcript types (HR: 0.782, 95% CI: 0.378–1.618, *p* = 0.2100). In relation to transcript type e13a2, e14a2 was also significantly more favorable when the Cox cure model was applied. The logistic part of the model led to an OR of 0.424 (95% CI: 0.197–0.912, *p* = 0.0282) and also the HR was significantly lower (HR: 0.595, 95% CI: 0.355–0.996, *p* = 0.0484). Using a multiple Cox cure model including transcript type and genotype and adjusting for other variables significant either in the logistic part (duration of TKI treatment) or the survival part (age at TKI discontinuation), the advantage of e14a2 over e13a2 in both parts of the model was confirmed. However, with the addition of transcript type, for genotype the picture of significance did not change: Compared with GG, the OR of GC for MMR loss was 0.439 (95% CI: 0.205–0.942, *p* = 0.0344) and the HR was 0.442 (95% CI: 0.175–1.119, *p* = 0.0850).

### Validation of pharmacogenotypes prognosis of molecular relapse-free survival probability

For the attempt of result validation, data on 103 Polish patients registered in the Polish STOP Imatinib database were received. Eight patients were excluded due to interferon-alpha pre-treatment and 2 due to missing data. With the same inclusion criteria as the patients in the learning sample, the 93 remaining patients constituted the validation sample. All Polish patients had only first-line imatinib before treatment stop. Baseline characteristics are listed in Table 1.

Out of 93 patients, 61 (66%, 95% CI: 55–74%) maintained MMR 6 months after imatinib stop. MMR maintenance proportion for genotype GC was 82% (95% CI: 67–91%), 85% (95% CI: 58–96%) for CC, and 44% (95% CI: 30–59%) for genotype GG. Again, the SNP rs460089 was associated with MMR maintenance after imatinib stop (*p* = 0.0009) with a significantly higher odds ratio (OR) for maintenance for GC genotype vs. GG (OR = 5.841, 95% CI: 2.097–16.269, *p* = 0.0007) and for CC vs. GG (OR = 7.027, 95% CI: 1.380–35.794, *p* = 0.0189). Using CC as reference group, the OR for MMR maintenance was not significantly different with GC genotype (OR = 0.831, 95% CI: 0.150–4.615, *p* = 0.8326).

In the Polish patients, only duration of DMR before imatinib discontinuation was significant (OR = 1.274, 95% CI: 1.021–1.588, *p* = 0.0319) when added to genotypes in multiple logistic regression analysis. Longer time of duration of DMR increased the probability to maintain MMR 6 months after imatinib stop while the overall association of the SNP rs460089 with MMR maintenance remained significant (*p* = 0.0011). The OR of GC vs. GG changed to 5. 998 (95% CI: 2.075–17.339, *p* = 0.009) and for CC vs. GG to 7.767 (95% CI: 1.473–40.939, *p* = 0.0157).

**Fig. 3:**
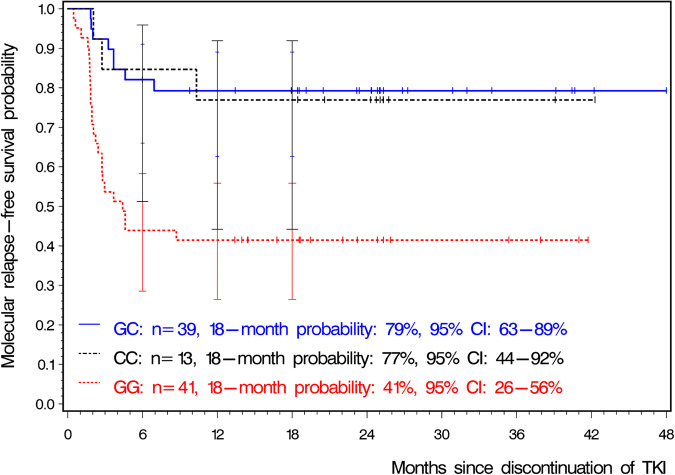
Probabilities of molecular relapse-free survival in 93 patients of the validation sample for each genotype of SNP rs460089. Bars at 6, 12, and 18 months indicate the upper and lower 95% CIs. CI confidence interval, TKI tyrosine kinase inhibitor. For the sake of consistency, the x-axis ended at 48 months. There was no event thereafter but 2 patients in the GC group were censored after 53.4 and 72.2 months.

Median observation time was 25 months. Minimum observation time for patients without molecular relapse was 6 months. Three patients experienced loss of MMR after 6 months. MRFS probability by 18 months was 62% (95% CI: 51–71%). Again, SNP rs460089 was significantly associated with MRFS probabilities after imatinib stop (*p* = 0.0011, Fig. 3). Patients with GC genotype had significantly higher MRFS probabilities compared with patients with GG (HR: 0.252, 95% CI: 0.113–0.561, *p* = 0.0007) but not with CC genotype (HR: 0.900, 95% CI: 0.239–3.395, *p* = 0.8769). In the validation sample, MRFS probabilities of genotype CC were higher than in the learning sample now resulting in a significant HR of 0.279 (95% CI: 0.084–0.929, *p* = 0.0376) for CC as compared with GG. In relation to genotype GG, GC and CC were overall also significantly more favorable when the Cox cure model was applied. The logistic part of the model led to an OR of 0.186 for GC (95% CI: 0.069–0.502, *p* = 0.0009) and to an OR of 0.213 for CC (95% CI: 0.051–0.890, *p* = 0.0340) expressing the lower proportions of MMR losses. In contrast, both HRs were not significantly lower (GC, HR: 0.617, 95% CI: 0.275–1.386, *p* = 0.2425; CC, HR: 0.341, 95% CI: 0.079–1.475, *p* = 0.1501) suggesting no obvious differences in the event time distributions between the genotype groups.

Using a multiple Cox cure model adjusting for other variables significant either in the logistic part (duration of DMR before TKI discontinuation) or the survival part (none), the picture did not change: Compared with GG, the OR for MMR loss for GC was 0.185 (95% CI: 0.067–0.512, *p* = 0.0012) and 0.199 (95% CI: 0.046–0.855, *p* = 0.0300) for CC. The HRs remained non-significant.

In the 93 patients of the validation sample, MMR maintenance at 6 months for transcript type e14a2 was 65% (95% CI: 51–76%), for the appearance of both transcript types 72% (95% CI: 49–88%) and 63% (95% CI: 43–79%) for e13a2. Overall, the association of transcript type with MMR maintenance at 6 months after TKI stop was not significant (*p* = 0.7919). Neither was transcript type significant when included in multiple logistic models. Considering loss of MMR as a time-to-event variable, again, transcript type had no significant influence on MRFS in any of the models.

## Discussion

Treatment-free remission / molecular relapse-free survival has become an important goal of CML therapy. Many clinical trials proved its clinical feasibility showing that about 40-60% of patients maintain DMR after TKI stop. TKI stopping is considered safe if the recommended requirements for TKI cessation are followed including frequent monitoring of measurable residual disease through standardized *BCR::ABL1* quantification [23].

Duration of TKI therapy and DMR before TKI stop are the most consistently reported prognostic factors for MRFS probabilities in many TKI cessation trials [16, 24–26]. Interferon-alfa pre-treated patients had higher MRFS probabilities after imatinib discontinuation [16, 27]. Immunoprofiles in CML patients seem to be relevant for remaining in TFR, exemplified by the association of the count and the more cytotoxic phenotype of natural killer cells with TFR probabilities [28, 29]. Interestingly, also the expression of the T-cell inhibitory receptor ligand CD86 on plasmacytoid dendritic cells affected the risk of molecular relapse after TKI stop [30]. Moreover, citing very recent works, it seems that DNA-based *BCR::ABL1* analysis is a promising molecular marker for the prognosis of TFR probabilities [31, 32].

Imatinib is the most used TKI in CML chronic phase patients. The response to imatinib is influenced by many factors including its bioavailability [33]. A poor response to imatinib is often associated with a sub-lethal intracellular concentration in CML cells. The intracellular concentration of imatinib is affected by patient adherence, absorption in the gastrointestinal tract, and distribution through the plasma membrane of the cells. In our previous work, multivariate analysis identified the regulatory SNP rs460089 located in the gene promotor region of drug transporter OCTN1 as being significantly associated with response in CML patients with first-line imatinib treatment [14]. The GC genotype was significantly associated with an optimal response by achieving durable MMR and DMR, while the GG genotype was related to a higher risk of imatinib failure.

This present work investigated in 176 EURO-SKI patients whether the genotypes have also prognostic impact on the maintenance of TFR (MMR) after (primarily) imatinib stop. Patients with GC genotype showed a significantly higher proportion of MMR maintenance after TKI stop than patients with GG genotype. A longer duration of imatinib treatment increased the probability of MMR maintenance after TKI cessation independently of the genotype. The superiority of the GC genotype over the GG genotype with respect to the maintenance of MMR was confirmed in 93 patients of the independent Polish validation sample. Here, it was longer duration of DMR that independently improved the probabilities of maintained MMR when added to genotype in a multivariable statistical model.

The more than 20% difference in the probabilities of MMR maintenance at 6 months in the EURO-SKI patient sample, 73% with GC (95% CI: 60–82%) and 51% with GG (95% CI: 41–61%), was clinically relevant and it was even more so with 38% in the Polish patient sample where MMR maintenance proportion for genotype GC was 82% (95% CI: 67–91%) and 44% (95% CI: 30–59%) for genotype GG.

The impact of the genotypes on the probability of TFR after treatment stop may be explained by the assumption that the GC genotype is associated with a sufficient intracellular concentration of imatinib allowing more efficient targeting of CML cells during the treatment compared to GG genotype.

Using the recessive model (GC + CC vs. GG), compared with the additive model, the results did not change significantly in any case, both in the EURO-SKI trial and the Polish sample. Several reasons support the additive model over the recessive model: The 6-month result of CC was closer to GG, and Fig. 2 clearly demonstrates that a 12- and 18-month probability difference of 21% between GC and CC would not justify combining the results of the two groups. Furthermore, combining GC with CC would result in a loss of information because the results of the GC and CC groups could no longer be distinguished. The specific contribution of GC to MMR maintenance in comparison to CC would become unknown. The significant difference between GC and GG would be diminished by adding the outcome of CC to GC, despite the similar results of GG and CC in the EURO-SKI trial. In the Polish patient group, the probabilities of MMR maintenance for genotype CC (85% at 6 months) were notably different from those in the EURO-SKI trial (61% at 6 months). This 24% difference, along with the limited reliability of the MMR maintenance estimation for CC in both samples (the width of the 95% CIs was around 40%), was well worth being explicitly shown. The dominant model (GG + GC vs CC) was not considered due to the small number of CC cases.

While the significant contribution of genotype in the multiple Cox cure model applied to the EURO-SKI sample was maintained, transcript type showed additional independent significance when added. However, in contrast to genotype, no significant influence of transcript type was observed in the validation sample.

The learning sample was a subsample of the EURO-SKI trial. While the genotype of the SNP rs460089 was only available in this subsample, in the EURO-SKI trial, the analyses of the influence of the durations of imatinib and of deep molecular response on MMR maintenance at 6 months were performed in 405 patients and the corresponding results should remain the reference for these two prognostic factors [16]. Based on our findings, a possible implication of genotyping the SNP rs460089 for CML patients starting with imatinib as first-line for clinical practice would require a next study. We assume that genotyping can be performed at the time of diagnosis or anytime during the imatinib treatment by using the fast and cheap TaqMan genotyping assay for the European population, or by direct sequencing for all ethnicities. In case of warning or failure 6 or 12 months after start of imatinib treatment in patients with GG genotype, a (timely) switch to a 2nd generation TKI could be considered. In patients with non-optimal response to imatinib and other genotypes than GG, we suggest to stick to the ELN recommendations for treatment management [23]. After the achievement of sustained DMR, imatinib (TKI) stop may be considered for patients with any genotype. Multivariable modeling suggested that longer imatinib treatment (longer duration of DMR) enhances the probability to maintain MMR at 6 months—independently of the genotype (Fig. 1). Similar to the conclusion drawn from the results of the EURO-SKI trial, the probabilities in Fig. 1 could support individual decisions of patients and physicians when trading the higher risk of molecular relapse due to an earlier treatment discontinuation against the (potentially) longer suffering from adverse reactions due to longer drug exposure.

In summary, the pharmacogenetics marker rs460089 showed prognostic influence on molecular relapse-free survival after TKI discontinuation in patients treated with first-line imatinib. Importantly, the result was validated in an independent patient sample. The prognostic significance was independent of and in addition to the established prognostic factors duration of imatinib treatment and duration of DMR before treatment stop. With regular evaluations and more data available, the SNP rs460089 may become part of a future TFR score including minimal duration of imatinib / TKI treatment or duration of DMR. Furthermore, a molecular mechanism of the significant impact of the SNP rs460089 on the outcome of CML patients treated with imatinib as first-line therapy and on TFR after TKI stop has yet to be experimentally explained and remains to be studied.

### Supplementary information

Supplementary material

Table S1

## Data Availability

The data that support the findings of this study are available from the corresponding author upon reasonable request.
