# Supplementary material for: The SNP rs460089 in the gene promoter of the drug transporter OCTN1 has prognostic value for treatment-free remission in chronic myeloid leukemia patients treated with imatinib
Source: Leukemia. 2023 Dec 21;38(2):318–25. doi: 10.1038/s41375-023-02109-2 (PMC10844071; doi:10.1038/s41375-023-02109-2)
Supplement: Supplementary file 1 — Supplementary material [file 41375_2023_2109_MOESM1_ESM.docx]

**Supplementary Methods**

The detailed version of the methods of the Article “The SNP rs460089 in the gene promoter of the drug transporter OCTN1 has prognostic value for treatment-free remission in chronic myeloid leukemia patients treated with imatinib” by Machova Polakova et al.

**Sequencing analysis**

Primers with sequences forward: 5´CAGTGCCTCCATGACTGTG3´ and reverse: 5´GCTGAGCAGGAAGAAGATGAG3´ were used to amplify the sequence containing rs460089 locus. Sequencing PCR was prepared using the FastStart™ High Fidelity PCR System (Roche Applied Science, Penzberg, Germany), PCR conditions : 95°C for 1min, 35 cycles of 95°C for 30s, of 62.2°C for 30s and 72°C for 1.0 min and 13s, finally 5 min hold at 72°C. Purification of amplicons was performed using the QIAquick PCR Purification Kit (Qiagen, Hilden, Germany), sequencing was using the BigDye® Terminator v3.1 Cycle Sequencing Kit (Thermo Fisher Scientific) (1.5 μl of purified PCR product + mixture containing 1 μl of 5x Sequencing Buffer, 1 μl of Big Dye v3.1, and 10pmol of the forward or reverse primer). The cycling conditions were: 25 cycles of 96°C for 10 s, 55°C for 10 s, and 60°C for 4 min, the sequencing PCR products were purified, dried resolved and denatured in formamide (Thermo Fisher Scientific). Sequencing of both strands was performed on the ABI PRISM 3500 (Thermo Fisher Scientific). Mutation Surveyor software v3.10 (Softgenetics; State College, PA, USA) was used for sequence analyses and SNP scoring.
