# Supplementary material for: The SNP rs460089 in the gene promoter of the drug transporter OCTN1 has prognostic value for treatment-free remission in chronic myeloid leukemia patients treated with imatinib
Source: Leukemia. 2023 Dec 21;38(2):318–25. doi: 10.1038/s41375-023-02109-2 (PMC10844071; doi:10.1038/s41375-023-02109-2)
Supplement: Supplementary file 2 — Table S1 [file 41375_2023_2109_MOESM2_ESM.docx]

| **SNP** | **Position in the genome** | **Alleles according to LDLink** | **Minor alelle** | **MAF (EUR)** | **D´** | **R^2^** | **Alelles in LD** | **Sequence according to dbSNP** |
| --- | --- | --- | --- | --- | --- | --- | --- | --- |
| **rs460089** | **chr5:131629772** | **(C/G)** | **C** | **0.3211** | **1.0** | **1.0** |  | **CCCTGCTGGGGTCTGGGCCCGGGGC[C/G]ACGCGGCCCGAGCAGATCGAGGGCC (fwd)** |
| rs270612 | chr5:131637338 | (G/A) | G | 0.3231 | 0.9954 | 0.9819 | **C**=G, **G**=A  fwd | GGATAAGGCCTCTTCCCCTGCTAAC[A/G]GGGAACTTCTGGGAGGCACTGAGAG (fwd) |
| rs270606 | chr5:131650867 | (A/G) | A | 0.3231 | 0.9954 | 0.9819 | **C**=A, **G**=G  fwd | TTCAGGCTTAGGGAGGATCGTCACA[C/T]GGAAGATGGATTCTGGGGACTTTGA (rev) |
| rs156322 | chr5:131653925 | (C/T) | C | 0.3231 | 0.9954 | 0.9819 | **C**=C, **G**=T  fwd | TTGAAATGTGCTACATGTCAGGGGA[A/G]TGATGGAAAGTACAATGCTTTTGAT (rev) |

**Table S1.** Identified SNPs in significant LD with the SNP rs460089

LDlink – a web tool for linkage disequilibrium analysis; dbSNP – the SNP databases; MAF (EUR)- Minor Allele Frequency in the European population; D‘= indicator of allele segregation for 2 genetic variants; the range of the values is 0-1 (values close to 1 indicate high linkage of alleles; R^2^ – correlation of alleles of 2 genetic variants within the range of the values 0-1 (values close to 1 indicate that allele of one genetic variant perfectly predicts allele of the second genetic variant)
